# Uncertain timing of reported acetaminophen ingestion in an adolescent analgesic overdose: a case report

**DOI:** 10.3389/fped.2026.1872986

**Published:** 2026-07-08

**Authors:** Wael A. Alghamdi

**Affiliations:** Department of Clinical Pharmacy, College of Pharmacy, King Khalid University, Abha, Saudi Arabia

**Keywords:** Acetaminophen, adolescent, overdose, TDM, uncertain ingestion

## Abstract

A 16-year-old male presented after reported ingestion of acetaminophen and multiple NSAIDs for dental pain. Although he reported taking acetaminophen 6,000 mg, his concentration was 22.3 mg/L approximately 19 h after the first reported ingestion, higher than expected from the reported dose and timing. Aminotransferases were normal, indicating no acetaminophen-induced liver injury at presentation. N-acetylcysteine was initiated because the concentration was clinically relevant in the setting of uncertain or staggered ingestion, and recovery was uneventful. This case highlights the importance of prioritizing measured acetaminophen concentrations and laboratory assessment over reported dose alone.

## 1 Introduction

Acetaminophen toxicity is one of the common causes of drug-induced liver injury and the second most common cause of liver transplantation worldwide (1, 2). The Rumack–Matthew nomogram remains the standard tool for guiding treatment in acute ingestions, and N-acetylcysteine (NAC) is the antidote of choice. However, the nomogram is less reliable in repeated or staggered ingestion, uncertain timing, or when the reported history is inconsistent with measured concentrations.

Non-steroidal anti-inflammatory drugs (NSAIDs), including diclofenac, ibuprofen, and meloxicam, are widely used analgesics. In overdose, they primarily cause gastrointestinal and renal effects, with hepatotoxicity being less common and usually idiosyncratic (3).

The case of a 16-year-old male who ingested acetaminophen together with multiple NSAIDs is presented. The measured acetaminophen concentration was clinically relevant and inconsistent with the reported acetaminophen 6,000 mg dose and timing, and NAC was initiated promptly. The educational value of this case lies in the discordance between a borderline reported acetaminophen dose, normal aminotransferases at presentation, and a clinically relevant acetaminophen concentration in the setting of uncertain timing.

## 2 Case presentation

A 16-year-old previously healthy male (weight 55 kg) presented to the emergency department with severe epigastric pain, nausea, and watery diarrhea. He denied vomiting, jaundice, bleeding, diaphoresis, or altered consciousness. There was no history of chronic illness, substance use, or prior overdose. History revealed self-medication for severe dental pain over the preceding two days. He reported ingesting approximately 26 tablets of diclofenac 46.5 mg (total 1,209 mg) between Thursday 00:00 (midnight) and Saturday 04:00. In addition, he consumed 12 tablets of acetaminophen 500 mg (total 6,000 mg) and 8 tablets of meloxicam between Friday 20:00 and Saturday 04:00. He also ingested two tablets of ibuprofen 600 mg (1,200 mg) at 03:00 on Saturday (Table 1). On arrival to the emergency department, approximately 60 h after the first reported diclofenac ingestion and 16 h after the first reported acetaminophen ingestion, the patient was alert, oriented, and vitally stable. He appeared well and non-icteric. Abdominal examination showed mild epigastric tenderness without organomegaly, and cardiovascular, respiratory, and neurological examinations were unremarkable.

**Table 1:** Timeline of reported ingestion, presentation, and management.

Time from first reported acetaminophen ingestion (hrs)	Event/Management	Findings/Labs
NA (Thu, 00:00)	Diclofenac ingestion reportedly began	—
0 h (Fri, 20:00)	Reported 12 acetaminophen 500 mg tablets (totaling 6,000 mg) and meloxicam 8 tablets (unknown strength) began over approximately 8 h	—
7 h (Sat, 03:00)	Reported ingestion of 2 ibuprofen 600 mg tablets (totaling 1,200 mg)	—
8 h (Sat, 04:00)	Last reported ingestion (APAP, meloxicam)	Epigastric pain, nausea, watery diarrhea
16 h (Sat, 12:00)	ED presentation (initial evaluation)	—
19 h (Sat, 15:00)	IV fluids; NAC initiated	APAP 22.3 mg/L
		INR 1.55
		ALP 309 U/L
		Total bilirubin 9.5 mg/dL
		ALT 6.1 U/L
		AST 24.1 U/L

Times are approximate and based on the patient's reported history. Acetaminophen-related timing is measured from the first reported acetaminophen ingestion at 20:00 on Friday.

ALP, alkaline phosphatase; ALT, alanine aminotransferase; APAP, acetaminophen; AST, aspartate aminotransferase; ED, emergency department; INR, international normalized ratio; IV, intravenous; NAC, N-acetylcysteine.

Initial venous blood gas demonstrated metabolic acidosis, which improved with intravenous fluids. Laboratory evaluation at approximately 19 h after the first reported acetaminophen ingestion showed an acetaminophen concentration of 22.3 mg/L. Coagulation studies revealed a prothrombin time of 20.9 s and an international normalized ratio (INR) of 1.55. Liver function tests demonstrated elevated alkaline phosphatase (309 U/L) and total bilirubin (9.5 mg/dL; with conjugated 1.79 mg/dL), while alanine aminotransferase (6.1 U/L) and aspartate aminotransferase (24.1 U/L) remained normal. Albumin was within the normal range (40.7 g/L), and gamma-glutamyl transferase was low (9.8 U/L). Renal function showed creatinine of 93.8 µmol/L and blood urea nitrogen of 1.43 mmol/L. Electrolytes revealed mild hypomagnesemia (0.73 mmol/L) and hyperphosphatemia (1.57 mmol/L), while sodium and potassium were normal. Complete blood count was within normal limits.

The patient was managed according to the standard three-bag intravenous NAC protocol: 150 mg/kg over 1 h, followed by 50 mg/kg over 4 h, and then 100 mg/kg over 16 h. The documented administered doses were 7,200 mg, 2,400 mg, and 4,800 mg, respectively. He also received intravenous fluids, intravenous ondansetron, and a proton pump inhibitor. During his hospital stay, his symptoms improved clinically and metabolic acidosis resolved after intravenous fluids. No additional laboratory values beyond those reported above were available for inclusion. He was later discharged in stable condition.

## 3 Discussion

This case illustrates the complexity of interpreting acetaminophen concentrations when the reported dose and timing are uncertain. The patient reported ingesting approximately 6,000 mg of acetaminophen, a dose below the commonly cited single-ingestion toxic threshold of 7,500 mg for adolescents and adults (1, 2, 4). However, his acetaminophen concentration was 22.3 mg/L approximately 19 h after the first reported acetaminophen ingestion, which was clinically relevant and inconsistent with the reported dose and timing.

As an exploratory comparison, the reported acetaminophen exposure was considered in relation to standard acetaminophen pharmacokinetic assumptions. Under a simplified single-dose, one-compartment oral pharmacokinetic assumption, with acetaminophen 6,000 mg taken at the earliest reported time, the expected concentration at approximately 19 h would be very low, generally below 1 mg/L (5, 6). However, this estimate should not be interpreted as a formal patient-specific pharmacokinetic prediction because the exact timing, distribution of doses, absorption, and bioavailability could not be confirmed. The measured concentration of 22.3 mg/L was therefore interpreted clinically as evidence of relevant acetaminophen exposure in the setting of uncertain history, rather than as proof of altered pharmacokinetics. However, analytical interference should also be considered. Hyperbilirubinemia has been reported to cause false-positive or falsely elevated acetaminophen concentrations, particularly with some enzymatic-colorimetric assays (7, 8). Because the patient had marked hyperbilirubinemia, a false-high acetaminophen result could not be fully excluded, and the measured concentration was interpreted cautiously in the broader clinical context. Formulation-related delayed absorption or delayed gastric emptying may also contribute to prolonged detectable concentrations. However, these possibilities would not exclude uncertainty in the reported dose, timing, or ingestion pattern, which remained central to the clinical interpretation in this case.

The patient did not have evidence of acetaminophen-induced liver injury at presentation, as aminotransferases were normal. Although he had a prolonged INR, elevated bilirubin, and metabolic acidosis, these findings are not typical early markers of acetaminophen hepatotoxicity in the absence of aminotransferase elevation. Because additional serial laboratory values were not available for analysis, these abnormalities could not be fully characterized over time or attributed to a single cause. They were therefore interpreted as concerning clinical abnormalities requiring monitoring, rather than evidence of established acetaminophen-induced liver injury.

NSAID co-ingestion may have contributed to the clinical presentation. The patient's epigastric pain, nausea, and watery diarrhea were consistent with NSAID-associated gastrointestinal toxicity, and fluid loss may have contributed to the metabolic acidosis that improved with intravenous fluids. Although renal function was not markedly impaired at presentation, mixed NSAID ingestion created a risk for renal injury, particularly in the setting of possible dehydration.

This case does not change established acetaminophen overdose management principles. Rather, it illustrates a practical teaching point: a reported acetaminophen dose below the usual single-ingestion toxic threshold and normal aminotransferases at presentation should not be falsely reassuring when ingestion timing is uncertain and the measured acetaminophen concentration remains clinically relevant.

The initiation of NAC was appropriate because the acetaminophen concentration was clinically relevant in the setting of uncertain or staggered ingestion, regardless of the reported acetaminophen 6,000 mg dose. Current recommendations support treatment when the concentration exceeds the applicable nomogram threshold in acute ingestion, and in repeated or uncertain ingestions when acetaminophen remains detectable at a clinically relevant concentration or aminotransferases are abnormal (2). This case highlights, consistent with established clinical practice, that a reported acetaminophen dose below 7,500 mg should not be falsely reassuring when timing and history are uncertain; a measured concentration may identify relevant exposure and justify standard NAC treatment and monitoring.

## 4 Conclusion

Acetaminophen concentrations are central to overdose management but must be interpreted in the context of reported dose, timing, and the reliability of the history. This case highlights the importance of prioritizing the measured acetaminophen concentration over reported dose alone when ingestion timing is uncertain. Early initiation of NAC is appropriate when acetaminophen concentrations exceed applicable treatment thresholds or remain clinically relevant in uncertain or staggered ingestion, even if the reported dose appears borderline.

## Data Availability

The original contributions presented in the study are included in the article/Supplementary Material, further inquiries can be directed to the corresponding author/s.
